# Thermal photonics for sustainability

**DOI:** 10.1515/nanoph-2024-0082

**Published:** 2024-02-29

**Authors:** Wei Li, Tianji Liu, Jia Zhu

**Affiliations:** GPL Photonics Laboratory, State Key Laboratory of Luminescence and Applications, 66498 Changchun Institute of Optics, Fine Mechanics and Physics, Chinese Academy of Sciences, Changchun 130033, China; University of Chinese Academy of Sciences, Beijing 100039, China; College of Engineering and Applied Sciences, 12581 Nanjing University, Jiangsu 210023, China

Thermal radiation represents a ubiquitous aspect of nature. As a main energy and entropy carrier, thermal radiation plays a fundamentally important role in a wide range of applications. In the emerging field of *thermal photonics*, using thermal photonic structures (at least one of the structural features are at a wavelength or sub-wavelength scale) can reshape thermal radiation to be drastically different from conventional thermal emitters, and offer exciting opportunities for energy applications [1], [2]. Thermal photonics is now emerging as a frontier in renewable energy research, with impacts on energy saving, carbon reduction and sustainable society. In the special issue “Thermal Photonics for Sustainability” of Nanophotonics, we collect and highlight a plethora of the latest development in thermal photonics and associated sustainability applications through reviews, perspectives, and research papers.

The review and perspectives contained in the issue provide comprehensive overview of nanophotonics-based radiative cooling. Yoo et al. reviewed the recent development of switchable radiative cooling strategy [3]. Based on different switching mechanisms such as wetting/drying, mechanical stimuli, thermochromic and electrochromic responses, self-adaptive thermal management is implemented to fulfill the requirement of stable temperature regulation. Zhang et al. exquisitely pointed out the problems and challenges when promoting radiative cooling from the laboratory to practice [4]. The perspective elaborates on theoretical constraints, the limitation of spectral selectivity, the difficulty of materials and structural design, nonuniformed evaluation protocols, commercialized problems, and possible solutions.

Daytime radiative cooling requires the spectral control of thermal radiation: concurrent high reflectance in the solar spectrum and high emissivity in the atmospheric windows [5]. Multilayer optical stacking, metamaterials, and photonic crystals were initially demonstrated for sub-ambient daytime radiative cooling [6], [7]. However, the design and fabrication complexity are not affordable for large-area industrial applications. Recently, polymeric and random photonic materials enable a high design degree of freedom and scalable manufacturing in nanophotonic control of thermal radiation [8]. Besides the enhanced spectral selectivity at the solar spectrum and atmospheric windows, more and more functionalities and industrial scalability are imparted to the emergent engineered photonic materials. In this special issue, Zhao et al. proposed a bilayer radiative cooling coating by covering TiO_2_/acrylic resin paint with a silica/poly(vinylidene fluoride-co-hexafluoropropylene) composite masking layer, exhibiting superhydrophobic property in a scalable fabrication process [9]. Han et al. developed recycled polymer-based passive radiative cooling materials, aiming at sustainable goal instead of using pristine polymer [10]. Combination of hydrophobicity, robust mechanical strength, durability, and scalability, Wang et al. presented a bilayer PDMS/nanoPE for efficient passive daytime radiative cooling [11]. Integrated with three-dimensional (3D) printing technology, Park et al. synthesized 3D printable hollow silica nanoparticles for sub-ambient daytime radiative cooling [12]. Kim et al. experimentally demonstrated polyacrylonitrile nanofibers (nanoPAN) based polymeric thermal radiator [13]. Enhanced bifacial PV performance is obtained with the combination of wavelength-selective scattering property of nanoPAN and metallic back reflector. Highlighting the adaptability with active cooling technologies for low-temperature enclosures, Yang et al. proposed a nanoporous polyethylene-based Janus type radiative cooling film, the conventional radiative cooling and the suppressed mid-infrared emission are obtained at top and bottom sides, respectively [14]. Felicelli et al. proposed a dual-layer structure composed of cellulose-based cotton paper and a thin BaSO_4_ nanoparticles-based coating, highlighting the radiative cooling performance and improved mechanical strength [15]. Catrysse and Fan demonstrated large-scale radiative cooling textiles for enhanced personal cooling using industrial scalable particle-free nonporous structured nonporous fibers that are fully compatible with standard textile production methods [16]. Dopphoopha et al. developed low thickness and eco-friendly aqueous paints composed of a Y_2_O_3_–ZnO double-layer design for enhanced scattering efficiency at ultraviolet and solar spectrum, respectively [17]. Tsang et al. proposed a bilayer architecture of thermal radiator composed of a porous poly(vinylidene fluoride-co-hexafluoropropene) top layer and a nanofibrous polytetrafluoroethene bottom layer, exhibiting near-unity long-wave infrared emissivity and near-ideal solar reflector concurrently [18].

Beyond static thermal emitters, dynamic thermal regulation provides self-adaptivity of thermal management against complicated and changeable temperature environment [19]. Liu et al. developed an electrochromic system based on reversible oxide electro-deposition/dissolution (ROE) of MnO_2_, enabling a bistable change between colored state and completely transparent and exhibiting large optical modulation depth and sufficient cycling stability [20]. Zhang et al. proposed a general framework to model a self-adaptive VO_2_ multilayer structure based on a temperature-doubler circuit, breaking the limitation of static photonic structures in energy harvesting from the diurnal cycle [21]. Liu and Zheng developed a reverse-switching phase-change materials based radiative cooling system, synchronizing with indoor air conditioning system to address issues of precise indoor temperature control [22]. In contrast to dense VO_2_ film and assisted with the Fabry–Perot resonant structures, Bhupathi et al. introduced a porous VO2/ZnSe/ITO/Glass optical thin film with enhanced visible transparency and improved infrared emissivity contrast for the application of thermochromic windows [23]. Li et al. theoretically investigated a VO_2_-based adaptive radiative cooling system with a high modulation rate of more than 0.9 [24]. The design of optical stacks is appropriate for a cost-effective and fault-tolerant fabrication process.

Directional thermal emission control has recently attracted substantial attention [25]. Angular response of thermal emitters is highly desirable for efficient and effective spatial thermal control. Shi et al. theoretically proposed a Weyl semimetal-based planar structure nonreciprocal thermal emitter, enabling ultra-broadband directional control for efficient thermal regulation [26]. Bae et al. analyzed and experimentally demonstrated Al_2_O_3_–Si_3_N_4_ epsilon-near-zero planar structures to directionally control the emissivity, which shows great potentials in radiative cooling on vertical surfaces [27]. Analogous to Salisbury screen, Sakar et al. developed a general design approach for the enhanced spatial and spectral control of thermal emitters, highlighting the directional emissions for concurrently dual polarizations in a three-layered planer heterostructures [28]. Gold et al. proposed a numerical gradient ascent (GAGA) algorithm for the optimization of one-dimensional magneto-optical photonic crystals, maximizing the nonreciprocal performance breaking Kirchhoff’s law in few-layer structures [29]. Wang et al. established a universal effective medium approach to achieving ultra-broadband directional thermal emission, the extraordinary spatial and spectral performance may have applications in high-efficiency information encryption and energy collection [30].

Lastly, beyond planar structures reported in this special issue, Yang et al. utilized bilayer twisted gratings to tailor the polarization state of thermal emission, full-covering Stokes parameters space has been numerically demonstrated via continuously changing the twist angle between grating layers [31]. In addition to far-field thermal emission control, Song et al. systematically investigated the performance evaluation of multi-junction-based near-field-thermophotovoltaic (NF-TPV) devices, offering quantitative guidance for the design of scalable NF-TPV devices and highlighting the significant role of multi-junction photovoltaic cells [32].

In summary, this special issue “Thermal Photonics for Sustainability” offers an extensive overview and introduces the latest development in thermal photonics, mainly covering scalable radiative cooling materials and structures, self-adaptive and dynamic thermal emission control, directional and broadband thermal emission, and full-Stokes polarization tuning and near-field thermophotovoltaic devices. We express our gratitude to all the authors and reviewers for their contributions.
